# The Importance of Trust in the Adoption and Use of Intelligent Assistive Technology by Older Adults to Support Aging in Place: Scoping Review Protocol

**DOI:** 10.2196/resprot.8772

**Published:** 2017-11-02

**Authors:** Josephine McMurray, Gillian Strudwick, Cheryl Forchuk, Adam Morse, Jessica Lachance, Arani Baskaran, Lauren Allison, Richard Booth

**Affiliations:** ^1^ Wilfrid Laurier University Waterloo, ON Canada; ^2^ Centre for Addiction and Mental Health Toronto, ON Canada; ^3^ Western University Arthur Labatt Family School of Nursing London, ON Canada

**Keywords:** assistive technology, trust, older adult, adoption, technology, aging in place, artificial intelligence, scoping review

## Abstract

**Background:**

Intelligent assistive technologies that complement and extend human abilities have proliferated in recent years. Service robots, home automation equipment, and other digital assistant devices possessing artificial intelligence are forms of assistive technologies that have become popular in society. Older adults (>55 years of age) have been identified by industry, government, and researchers as a demographic who can benefit significantly from the use of intelligent assistive technology to support various activities of daily living.

**Objective:**

The purpose of this scoping review is to summarize the literature on the importance of the concept of “trust” in the adoption of intelligent assistive technologies to assist aging in place by older adults.

**Methods:**

Using a scoping review methodology, our search strategy will examine the following databases: ACM Digital Library, Allied and Complementary Medicine Database (AMED), Cumulative Index to Nursing and Allied Health Literature (CINAHL), Medline, PsycINFO, Scopus, and Web of Science. Two reviewers will independently screen the initial titles obtained from the search, and these results will be further inspected by other members of the research team for inclusion in the review.

**Results:**

This review will provide insights into how the concept of trust is actualized in the adoption of intelligent assistive technology by older adults. Preliminary sensitization to the literature suggests that the concept of trust is fluid, unstable, and intimately tied to the type of intelligent assistive technology being examined. Furthermore, a wide range of theoretical lenses that include elements of trust have been used to examine this concept.

**Conclusions:**

This review will describe the concept of trust in the adoption of intelligent assistive technology by older adults, and will provide insights for practitioners, policy makers, and technology vendors for future practice.

## Introduction

Technologies that amplify or augment human abilities have matured and proliferated in recent years. Robotics, home automation, and other forms of mobile systems are examples of technologies that assist human users in tasks, and have become subtly commonplace in people’s lives. While the term *assistive technology* has been historically aligned with devices used by individuals with physical or cognitive deficits (eg, wheelchairs, speech-generating devices [1]), newer forms of assistive technologies possessing elements of artificial intelligence have emerged in recent years. The term *intelligent assistive technologies* includes a broad range of technological systems used to support or extend human abilities [2], and are labelled as *smart* or *intelligent* due to their ability to sense and respond to user needs and a changing environment *.* The term is also used to describe a platform capable of operating autonomously or within larger networks of related devices [3]. Due to the intelligent nature of these devices, they can be used to support human users with a multitude of everyday tasks, such as household cleaning and maintenance, transportation, food and meal preparation, and various self-care management and recreational activities. Examples of intelligent devices relevant to older adults include autonomous vehicles, sensor-driven home emergency response systems, and personal robots [3].

While once only used for basic sensory and detection purposes [4], the consumer-level scaling of current generation smart and intelligent technology has produced systems that support more nuanced and complex aspects of people’s daily living [3,5]. Home automation [6], service robots [7], wearable biometric sensors [8], relational companion agents [9], and *Internet of Things* applications [10] are now available to consumers to support a multitude of tasks, both in the home and the workplace. While there is broad societal interest in the use of these forms of smart technologies, they are increasingly being used to assist older adult populations (>55 years of age) to age in place [6,10-15]. With the global population of people over 65 expected to triple by 2050 [16], it has been proposed that assistive and other forms of artificially intelligent technology may play helpful roles in the early detection and management of age-related conditions [3]. Furthermore, by enabling older adults to *age in place* or, “live in one’s own home and community safely, independently, and comfortably, regardless of age, income, or ability level” [17], older adults will be able to remain supported in their home and community, and potentially delay the use of institutional care [3]. Older adults who experience physical, mental, or cognitive deficits have been specifically identified as people who could benefit from these forms of intelligent assistive technologies to support their aging in place [6,18]. For instance, there has been a steadily growing body of literature exploring the use of these forms of intelligent assistive technologies to support the early detection and management of a variety of conditions, including Alzheimer’s disease and dementia [19]. Other potential user groups, such as those experiencing mental health challenges [20], visual or auditory impairments [21], or medically fragility [22] have all been explored by researchers as conditions that can be supported through the appropriate use of intelligent assistive technology.

Over the last decade, a sizable body of research has been generated exploring the use of intelligent assistive technology in older adult populations. Topics include a diverse range of intelligent assistive technology, including devices that accomplish isolated or specific tasks like vacuuming, cleaning, and remote lighting control [12,23-26]. More recently, personalized companionship, virtual presence, virtual assistant, and sensory technologies that offer customized lifestyle supports based on various socio-contextual inputs (ie, diet, sleep, activity, time of day) have also become popularized [10,26]. Some intelligent assistive technologies also possess the ability to leverage multimodal interaction between other distinct smart devices to surround an individual with personalized support. For instance, Amazon Echo (a conversational virtual assistant device) can help a human user complete tasks via voice commands (eg, create shopping lists, provide news/traffic information) [27]. The Echo assistant can also control other home automation technology (eg, smart lighting, cleaning service robots) to generate an interlinked, intelligent network of assistive technology around a human user [27]. Given the autonomous nature of some intelligent assistive technologies, researchers are interested in exploring the possibility of personalized, fluid, human-technical relationships with these devices [28-31].

As assistive technologies acquire and demonstrate intelligence through their functionality, human users may begin to perceive them more like autonomous agents [32]. Due to this perception, human users assume more risk as they move from predictable and controllable interactions with nonintelligent devices (ie, traditional assistive technology) to intelligent devices where responses and outcomes may become less predictable. One concept noted to be important in the building of this kind of human-technical relationship is *trust* [33] *.* Trust is a construct we assign to a human’s willingness to rely on the actions of an autonomous agent (ie, intelligent assistive technology), whose behaviors are not directly controlled by an individual [34]. To date, the concept of trust regarding older adults’ adoption of intelligent assistive technology has been examined directly or indirectly through a variety of fashions, including: (1) whether human users felt comfortable following the directions of an intelligent assistive technology [29,31,35]; (2) if the technology espoused a perceivable feeling of trustworthiness [31]; and (3) if the technology, in its function or presence, became a trusted technological entity [36]. While numerous articles have addressed various constructs associated with the role of trust in older adults’ adoption of technology [23,29,31,35,36], there has yet to be a broad search and summary of current literature in the domain.

The increasing embeddedness and omnipresent nature of many intelligent assistive technologies suggests that a review of the state of the research in the domain is timely. To address this gap, we describe the protocol for a scoping review that will seek to explore the concept of trust in relation to older adults and their adoption and use of intelligent assistive technologies that support aging in place.

## Methods

### Scoping Review

Scoping reviews are commonly undertaken, “to examine the extent, range and nature of a research activity” [37] in a given domain, especially, “when it is difficult to visualize the range of material that might be available” [38]. A scoping review methodology using the approach advised by Arksey and O’Malley [38] and advanced by Levac et al [37] was selected over more time-intensive synthesis approaches (ie, systematic review, meta-analysis).

#### Step 1: Identification of the Research Question

Levac et al [37] recommend that research questions for scoping reviews be “broad” in nature, but linked with a specific and, “clearly articulated scope of inquiry.” Using these conceptual recommendations, the operational research question to be used in this review was derived through research team discussion: “How important is *trust* to the adoption and use of intelligent assistive technology that allows older adults to age in place?”

#### Step 2: Identification of Relevant Studies

In this scoping review we will draw upon a variety of scholarly reference databases, including: ACM Digital Library, Allied and Complementary Medicine Database (AMED), Cumulative Index to Nursing and Allied Health Literature (CINAHL), Medline, PsycINFO, Scopus, and Web of Science. We will also capture grey literature through Dissertation Abstracts and Google Scholar searches. Academic literature generated over the last decade (2006-2017) will be targeted, given the rapid evolution of technology in this research domain. Through consultation with a health sciences librarian, research team meetings, and exploration of other review publications on similar topics, search syntax and inclusion/exclusion criteria were developed (Textbox 1). The search syntax targets keywords related to the three major variable components comprised in the research question: (1) older adult population, (2) the intelligent assistive technology, and (3) the concept of trust. The search syntax included “older adult*” OR “senior*” OR “baby boomer*” OR “aging in place” OR “elder*” AND “robot*” OR “assistive technolog*” OR “technolog*” OR “smart technolog*” OR “artificial intelligence” OR “intelligent technolog*” OR “intelligent assistive technolog*” AND “trust*” OR “reliab*” OR “engage*” OR “adopt*” OR “accept*” OR “depend*” OR “confiden*” OR “rely”. The search syntax will be run in each of the reference databases, and results will be extracted as RIS files and saved separately. All results arising from the reference databases will be imported into the Covidence (Veritas Health Innovation) software platform for review.

**Inclusion and exclusion criteria for the scoping review.**

Inclusion criteria

- English language research papers and conference abstracts
- 2006 to 2017
- Aged >55 years and above, or defined otherwise as older adult, elderly, or senior
- Research that examines intelligent assistive device use by older adult population for some activity to support *aging in place*, related to activities of daily living, health, or physical/mental behaviors (aged >55 years, or defined otherwise)
- Quantitative and qualitative research studies

Exclusion criteria

- Book reviews, textbooks chapters, opinion papers, and commentaries
- Intelligent assistive technology/devices examinations that lack “intelligent” definition (eg, magnification books, wheel chairs without digital brains)
- Intelligent assistive technology/devices that are used to support other activities (eg, farming, employment)
- Aged <54 years, if not defined by authors as being older adult, senior, or elderly
- Nonresearch studies

#### Step 3: Selection of Studies

Two members of the research team (AM, JL) will independently screen all retrieved titles and abstracts for applicability to the review’s research question. Discrepancies resulting from the independent initial screen will be discussed between the two research team members until consensus is reached. After this initial independent screening, other members of the research team (RB, JM) will review the full-text articles of short-listed results and, as suggested by Levac et al [37], develop a post hoc inclusion and exclusion criteria that will be applied to the short-list.

#### Step 4: Extracting Data From Studies

A preliminary researcher-developed data charting tool has been generated to facilitate the extraction of data from the studies (Table 1). Levac et al [37] suggest that both the process of extracting data from studies and the development of the charting tool should be an iterative, data-driven process, whereby increased familiarity with the study findings reciprocally assists in developing/refining the charting tool. Using a descriptive analytical method, findings emerging from each study will be qualitatively summarized in a narrative fashion, and common themes and results will be identified. Through this process, it is expected that the charting tool will evolve as data is extracted from the respective studies, and important findings related to the research question (not *a priori* conceptualized) are added to the data fields.

**Table 1:** Data extraction table.

Data	Details for extraction
Article information	Author
	Year
	Title
	Journal
	Abstract
	Study type
	Theoretical/conceptual model
Population	Definition of older adult
	Inclusion criteria
	Research setting location
Intervention	Description/type of intelligent assistive technology or system
	Measures (if applicable)
Trust	Operational definition of *trust*
	Measure or qualitative aspects relevant to *trust*
Study findings	Brief overall study findings description

#### Step 5: Collating and Summarizing Data

To better organize the synthesis findings, we will use tables to help accentuate the abstracted themes. Frequency data related to a numerical count of studies that explore specific themes or elements related to the research question will be calculated. Similarly, tables will also provide a clear and concise visualization of subthemes and issues arising from the main synthesis findings. Both NVivo 11 (QSR International) and Microsoft Excel (Microsoft Corp) software applications will be used to assist in coding and quantifying emergent findings from the studies. Most of the findings will be reported in a narrative fashion, qualitatively describing the current state-of-the-art in terms of trust and older adults’ adoption/use of intelligent assistive technology. Drawing methodological insights from the constant comparative technique [39], all study findings will be constantly compared against other findings in an effort to develop a cohesive thematic description of the state-of-the-art regarding the research question.

## Results

This review will provide insights into how the concept of trust is actualized in the adoption of intelligent assistive technology by older adults. Preliminary sensitization to the literature suggests that the concept of trust is fluid, unstable, and intimately tied to the type of intelligent assistive technology being examined. Furthermore, a wide range of theoretical lenses that include elements of trust have been used to examine this concept.

## Discussion

### Preliminary Findings

To date, we have generated some preliminary insights into the concept of *trust* in the adoption of intelligent assistive technology by older adults to support aging in place, through an initial search of literature in the domain to derive sensitizing constructs for the review team. Sensitizing constructs provide, “a starting point for a qualitative study” [40]. First, the vernacular in this area of inquiry is diverse and unstable. The various uses of neologisms and other specific terms to denote various typologies of intelligent assistive technology is remarkable, and will likely become a finding that is embedded into the data extraction tool. Second, given the nonspecificity of the search algorithm, a broad range of intelligent technologies will likely be identified in the research domain over the last decade. Smart assistive living environments, ambient assisted living, social robots, virtual presence, and personalized cloud technologies (among others) have all been featured in the sensitizing literature. Third, the operational definition of the older adult population included in this study has varied significantly in other studies, ranging from 50 [41] to 90 years of age [42]. Finally, conceptualizations of trust also appear to vary significantly between studies. A range of different theoretical perspectives have commonly been used to operationalize the measurement of the concept of trust, including the Technology Acceptance Model [43], Systems Engineering Initiative for Patient Safety [33], and other sociological frameworks [34]. Consequently, the concept of trust has also been defined in a variety of ways, often related to the functional features of the technology, such as its usefulness, learned nature, or some element of situational presence with a human user [44-46].

### Limitations

A potential limitation of this scoping review will be the lack of an explicit quality assessment of the included articles. The lack of a quality assessment review of the articles is common in scoping review methodologies, as the primary goal of this type of review is to generate rapid insights into an emerging domain to inform future inquiry. Furthermore, given the primordial state of research in this area (ie, no randomized controlled trial studies were found in any of the preliminary scan of articles arising from the search syntax), applying a formalized quality assessment component to this review may inadvertently exclude pilot studies and other research designs that produce findings with constrained external validity, or those that are qualitative in nature.

### Conclusion

As a research domain that will undoubtedly become more complex and diverse in the coming decade, it is essential that stakeholders have a deeper understanding of the construct of *trust* in relation to older adults and their adoption and use of intelligent assistive technology. To date there has been no formative research synthesis exploring the concept of trust in the adoption of intelligent assistive technology by older adults. This review will provide a range of stakeholders with a better understanding of how these forms of intelligent technologies can serve older adult populations. Furthermore, the review will assist with appropriate scaling of these innovations to address the specific adoption needs of the older adult population in what can be an unfamiliar technological landscape. We are confident that our review will generate useful preliminary insights into how trust must be factored into older adults’ adoption of intelligent assistive technology to support their aging in place. Finally, our development of a detailed data extraction tool will provide the groundwork for future explorations of the role of trust in the adoption of intelligent technologies in other consumer populations (eg, youth/adolescents, expectant mothers, parents).

## Appendix

Multimedia Appendix 1 Reviewer scores from review of SSHRC grant.
