# Supplementary material for: The Importance of Trust in the Adoption and Use of Intelligent Assistive Technology by Older Adults to Support Aging in Place: Scoping Review Protocol
Source: JMIR Res Protoc. 2017 Nov 2;6(11):e218. doi: 10.2196/resprot.8772 (PMC5691240; doi:10.2196/resprot.8772)
Supplement: Multimedia Appendix 1 [file resprot_v6i11e218_app1.pdf]

Subject: RE: SSHRC KSG - feedback from competition
From: "Conlon,Rachel" <Rachel.Conlon@sshrc-crsh.gc.ca>
Date: 2017-05-30, 13:43
To: "'rbooth5@uwo.ca'" <rbooth5@uwo.ca>
CC: Knowledge Synthesis Grant <ksggl-sscgl@sshrc-crsh.gc.ca>

Dear Richard,

While we don't collect written comments from the merit reviewers, I can provide you with your average scores in the competition. Each score is out of six.

Challenge 4.50/6
Feasibility 4.80/6
Capability 5.15/6

For a total score of 14.36.

The last funded application received a score of 14.19

I hope this helps,

Sincerely,

Rachel Conlon
Senior Program Officer/Agente de programme principale
Office of the Associate Vice-President. Future Challenges
Bureau de la vice-présidente adjointe, Défis de demain
Social Sciences and Humanities Research Council of Canada
Conseil de recherches en sciences humaines du Canada
350 Albert Street | 350, rue Albert, Ottawa, ON K1P 6G4
E | C : rachel.conlon@sshrc-crsh.gc.ca
T : 613.944.5327 **F | T :** 613.943.1329
www.sshrc-crsh.gc.ca

Stay Connected *Restez branchés*

From: Richard Booth [mailto:rbooth6@uwo.ca]
Sent: Tuesday, May 30, 2017 12:44 PM
To: Conlon,Rachel
Subject: SSHRC KSG - feedback from competition

Hello Rachel,

I hope this message finds you well. I am one of the current KSG holders and enjoyed the SSHRC event May 18th in Ottawa, and pulled your email address from a message you sent
